# Female Genital Mutilation: perceptions of healthcare professionals and the perspective of the migrant families

**DOI:** 10.1186/1471-2458-10-193

**Published:** 2010-04-13

**Authors:** Adriana Kaplan-Marcusán, Natividad Fernández del Rio, Juana Moreno-Navarro, Ma José Castany-Fàbregas, Marta Ruiz Nogueras, Laura Muñoz-Ortiz, Eliana Monguí-Avila, Pere Torán-Monserrat

**Affiliations:** 1 Department of Social and Cultural Anthropology, Autonomous University of Barcelona, 08193 Bellaterra (Barcelona), Spain; 2 Primary Healthcare Centre Mataró 6 (Gatassa), Catalan Health Institute, Camí del Mig 36 (4a planta), 08303 Mataró (Barcelona), Spain; 3 Primary Healthcare Centre Sant Andreu de Llavaneres, Catalan Health Institute, Passeig Jaume Brutau s/n, 08392 Sant Andreu de Llavaneres (Barcelona), Spain; 4 Primary Healthcare Research Support Unit of Barcelonès Nord i Maresme, IDIAP Jordi Gol, Camí del Mig 36 (3a planta), 08303 Mataró (Barcelona), Spain; 5 Corporación Mesa Mujer y Economía, Universidad Nacional, Bogotá, Colombia

## Abstract

**Background:**

Female Genital Mutilation (FGM) is a traditional practice which is harmful to health and is profoundly rooted in many Sub-Saharan African countries. It is estimated that between 100 and 140 million women around the world have been victims of some form of FGM and that each year 3 million girls are at risk of being submitted to these practices. As a consequence of the migratory phenomena, the problems associated with FGM have extended to the Western countries receiving the immigrants. The practice of FGM has repercussions on the physical, psychic, sexual and reproductive health of women, severely deteriorating their current and future quality of life. Primary healthcare professionals are in a privileged position to detect and prevent these situations of risk which will be increasingly more present in Spain.

**Methods/Design:**

The objective of the study is to describe the knowledge, attitudes and practices of the primary healthcare professionals, working in 25 health care centres in Barcelona and Girona regions, regarding FGM, as well as to investigate the perception of this subject among the migrant communities from countries with strong roots in these practices. A transversal descriptive study will be performed with a questionnaire to primary healthcare professionals and migrant healthcare users.

Using a questionnaire specifically designed for this study, we will evaluate the knowledge, attitudes and skills of the healthcare professionals to approach this problem. In a sub-study, performed with a similar methodology but with the participation of cultural mediators, the perceptions of the migrant families in relation to their position and expectancies in view of the result of preventive interventions will be determined.

Variables related to the socio-demographic aspects, knowledge of FGM (types, cultural origin, geographic distribution and ethnicity), evaluation of attitudes and beliefs towards FGM and previous contact or experience with cases or risk situations will be obtained.

**Discussion:**

Knowledge of these harmful practices and a preventive approach from a transcultural perspective may represent a positive intervention model for integrative care of immigrants, respecting their values and culture while also being effective in eliminating the physical and psychic consequences of FGM.

## Background

In the last decades, in Spain and other Western countries there has been a distorted visualisation of the phenomena associated with gender and immigration, especially in the case of sub-Saharan women, their daughters and female genital mutilation (FGM) [1,2]. The polarised reactions that they generate in the society of destination do not help to change the conditions of life and health of the girls but rather their integration is presented in dichotomic terms: assimilation versus stigmatisation or criminalisation [3,4].

The WHO calculates that between 100 and 140 million women and girls in the world have been victims of some kind of FGM. It has also been estimated that each year about three million girls are at risk or are subjected to some kind of ablation, essentially in the 28 countries in sub-Saharan Africa, where this type of ritual has strong, ancestral roots. Since 1952, when the UN Human Rights Commission adopted the first resolution on this matter, most international organizations have spoken out against these harmful traditional practices, encouraging their state members to promote legislative initiatives to eradicate these practices. In spite of all the efforts by international organizations and governments, we are faced with a problem which, as mentioned in a report by the UNICEF Innocenti centre, "continues to be one of the most persistent and omnipresent violations of human rights, and which is also tolerated in silence" [5,6].

FGM is carried out in girls aged between birth (7 days) and pre-adolescence (10-12 years), always before the first menstruation and marriage.

According to the WHO [6] four types of FGM can be distinguished according to the severity of the intervention practised: Type I or clitoridectomy, partial or total removal of the clitoris and/or the prepuce; Type II or excision, partial or total removal of the clitoris and the labia minora, with or without excision of the labia majora; Type III or infibulation, narrowing of the vaginal orifice with creation of a covering seal by cutting and appositioning the labia minora and/or the labia majora, with or without excision of the clitoris. A small hole is left to allow for urinating and menstrual bleeding; and Type IV which includes various practices in the genital area, with a varying degree of severity and without any therapeutic purpose (piercing, dry sex, stretching, cauterising the clitoris, etc).

Types I and II are predominant in Western and Central sub-Saharan African countries, whereas type III is the most common form of FGM in Eastern Africa. It should be specified that not all African countries practice FGM, and neither is it practiced by all the ethnic groups in one and the same country. It is pre-Islamic and is practiced by Muslims, Coptic Christians and Falasha Jews.

The performance of FGM has an impact on the physical, psychic, sexual and reproductive health of women, severely deteriorating their present and future quality of life [7-13].

The social and cultural roots of these practices condition their perpetuation, despite important restrictive legal initiatives having been developed in some countries of origin and destination of the immigrants.

In addition to the many initiatives of the international agencies, who recommend their prohibition (WHO, UNICEF, UNFPA, EU), FGM in any of its forms is considered as a crime of injury in Spain, being described and punished in article 149 of the Penal Code with sentences of 6 to 12 years in prison even if the mutilation has been performed abroad.

On the other hand, the lack of intervention by healthcare professionals to avoid genital mutilation in situations of risk is considered to be a crime of omission of duty to avoid and promote persecution in article 450 of the Spanish Penal Code. This means, for example, that professionals who, in relation to their work activity, are aware of FGM having been performed or of a girl at risk of being submitted to this practice and do not avoid or report the event, are committing a crime which may be punished with a prison sentence.

In Spain, the first cases were detected in Catalonia in 1993 and were discovered and reported by healthcare professionals. These cases were reported and resulted in a verdict of not guilty for the parents of the girls who claimed "no intention to injure and error of prohibition". Since then there is no knowledge of further mutilations having taken place in Spain, although it is known that the families take advantage of their holidays to the country of origin to carry out the initiation of their daughters. In some cases in which the environment of the family of the minor was investigated, the parents declared than FGM had been carried out abroad (in their country of origin) believing that Spanish law could do nothing. The most common form of presentation of the problem associated with FGM in our setting is a comment made by a family with daughters of an age of initiation, concerning an imminent journey to Africa. This situation is mainly detected by healthcare professionals, social services and schools. Judicial actions undertaken in view of knowledge of a journey of girls at risk in their country of origin consist in preventive withholding of the passport and the commitment of the parents to not mutilate their daughters, in addition to an obligatory check up on their return.

In the case of FGM there is a complex interaction between rights, values and cultures which indicate that we can not be limited to only applying punishment. Some of the control measures proposed may harm the intimacy and dignity of the minors to be protected (such as the obligation of undergoing gynaecologic check up until the age of 18) or may lead to a serious family destructuration on withdrawal of the custody of the parents and causing the girls to be admitted under the protection of social services. All of this generates professional doubts and dilemmas with respect to the consequences of intervention (reporting, court intervention, family disintegration and destructuration, criminalization of the parents, possibility of no return of the minors for fear of the legal consequences in the country of destination) or no intervention (professional consequences of omission, irreparable physical and psychic lesions in the girls).

There are few studies in Spain related to FGM in primary care. A previous survey carried out by investigators of our team of healthcare professionals in the county of the Maresme (Barcelona) [14,15] reported that 56% of those surveyed did not correctly identify the typology of FGM, 17% were not interested in the subject and their answer to the question "What would you do in the case of FGM?" was: "ignored". Twenty-one percent of the personnel of the sexual and reproductive healthcare programme, 7% of the paediatricians and 5% of the general medicine professionals declared having detected or known of some case in both mothers and daughters. This study demonstrated the profound lack of knowledge of the healthcare professionals in relation to this subject, raising the need to sensitise and educate from the privileged setting of primary healthcare, where most of the cases or situations at risk are detected. In other European countries the situation is similar [16-18].

The important migratory waves of sub-Saharan populations to the European Union in the last years are shaping societies which are increasingly more complex and diverse. The way to deal with the health problems affecting these people represents a challenge for the healthcare systems [19] and for the professionals working within who must develop approaches to allow transcultural care [20-22].

Based on the production of mapping FGM in Spain, with data of the census and municipal registration, updated in 2005 we have estimated that 3,659 women living in Catalonia have undergone some type of FGM; and 2,189 girls are at risk of having some type of FGM performed in the next years, without taking into account the impact of the last processes of immigration regularisation.

The deep-rooted social and cultural nature of these practices conditions their perpetuation despite the development of important legal restrictive initiatives in some countries of origin and destination of the immigrants. However, when migrant women return to their societies of origin they are points of reference, credibility and recognition in such transcendent aspects as reproductive health and education [23]. These women bear new knowledge acquired in Europe which has been incorporated and put into practice during the migratory process. Thus, the exploration of the beliefs and perceptions of the sub-Saharan immigrants attending our healthcare centres in relation to FGM may be of interest.

In addition to an adequate legislative framework, a preventive standpoint is essential which, with knowledge and sensitisation, will allow healthcare providers to approach the question of FGM and thereby avoid the conflicts brought about by legislation of aspects linked to the privacy and identity of the migrant persons.

Given the accessibility of the population to the universal healthcare available in Spain, primary healthcare may have a key role in the prevention of FGM [24,25], with these professionals being in the first line for dealing with this problem. Interdisciplinary teams with paediatricians, family physicians, nurse, social workers, gynaecologists and midwives, allow an integral approach to the families from the countries of sub-Saharan Africa where the question of FGM is always very present [26,27].

We are not aware of other previous experiences developed in primary healthcare in Spain or in Europe. Several studies have described registries of cases and others have reported knowledge, attitudes and practices related to FGM by professionals of specialised care [28], but to our knowledge there are no publications on these aspects in relation to first level healthcare or to preventive work undertaken from the primary care setting. We therefore developed the present study to provide knowledge and elements for improvement in the approach to FGM to prevent this problem on behalf of the healthcare professionals.

### Objectives

#### Main objectives

1. Describe the knowledge, attitudes and practices of the professionals of the primary healthcare teams related to the practice of FGM and how to approach any of the situations related to this procedure.

2. Know the degree of knowledge the primary care professionals have on the regulatory legislative framework in relation to the FGM and the penal consequences for both the migrant families and the healthcare professionals.

3. Identify dilemmas of ethics and interventions given the hypothetical presentation of a problem related to FGM in any of its presentations (girls at risk, recent FGM, knowledge of intention to perform FGM).

#### Secondary objectives

4. Identify the training needs of the primary care professionals in relation to how to approach FGM.

5. Quantify the frequency of presentation of problems related to FGM in primary care offices.

6. Attempt to approach the position of the immigrant families given the intervention of healthcare agents to avoid these practices.

## Methods/design

This is a transversal, descriptive study based on a survey regarding the knowledge, attitudes and practices (KAP) of the professionals, and users from countries of sub-Saharan Africa versus the performance of FGM. The study will be carried out in the 25 Primary Healthcare Centres from (PHC) Northern Barcelona and the Maresme Region (Barcelona, Spain) and from the counties of Girona (Girona, Spain) providing healthcare coverage to a population of 487,000 inhabitants.

### Study subjects. Sample size and selection method

#### Healthcare Workers

Healthcare providers of the teams of paediatrics, social work, family medicine, nursing and gynaecology-obstetrics associated with the PHC of Northern Barcelona, the Maresme and the counties of Girona.

#### Selection of healthcare providers

The study includes all the professionals of the PHC teams, the Sexual and Reproductive Healthcare Programme and the Departments of Paediatrics and Gynaecology-Obstetrics of the hospital centres. It will also include the social workers linked to PHC. In the Catalonian Healthcare System, primary healthcare professionals work in teams who attend the healthcare needs of the population assigned in a determined territory. These teams are made up of family physicians, general medicine physicians, paediatricians, nurses and social workers. Support programs are available within the setting of sexual and reproductive health and these are mainly constituted by gynaecologists, nurses and midwives. All these healthcare providers will be asked to voluntarily and anonymously participate in filling out the questionnaire.

The target population will therefore be all the healthcare providers of the previously mentioned groups and teams who will be contacted through the heads of the teams and informative sessions in each of the centres and departments. A response rate of around 75% has been estimated, similar to that obtained by the same system in a previous study by our group in 2001 and 2004 [14,15].

#### Immigrant families

The questionnaire aimed at families from countries of sub-Saharan Africa in which FGM is present and who use the healthcare centres will be given to the group of men and women of the community who, through the intervention of cultural mediators, agree to participate in the study and who have been directly or indirectly in contact with the problem. In this part of the study we cannot perform a randomised or systematic sample, since no randomisation list is available, but rather we must base the sample on criteria of accessibility (temporal, idiomatic, cultural, opportunity, etc.) to these mediators to at least allow determination of the point of view of the persons affected from the community, previously assuming the possibility of introducing a certain bias in selection. A convenience sample will be made according to attendance to the healthcare centre and fulfilment of the inclusion criteria: to be a legal resident in Spain during the last year, be over the age of 18 years, attend the primary care centre for any reason and be from any of the countries in which FGM is practised. Two cultural mediators from the community (Senegal and Gambia) who have undertaken specific training in intercultural mediation and who usually provide such mediatory support to the PHC will participate in these surveys. They will receive specific instruction regarding the objectives of the study, the content of the questionnaires and the clarifications or explanations which may be given to the participants. Prior to beginning the field work, 3 to 6 simulated interviews will be performed in the presence of one of the members of the investigative team. The work of the mediators will be fundamentally based on introducing the interviewers and accompanying them throughout the whole interview process to clarify any concept or doubt which may emerge during the interview. Translation will also be provided by these mediators, if necessary.

Informed consent will be requested from participants in the study. In the case of migrant people, cultural mediators may be required to obtain this informed consent.

Sample size: According to the last municipal registry data available (11% of the immigrant population, 13.5% of which is of sub-Saharan origin), the population eligible in our study area is of 7,200 individuals. We assume that the prevalence of FGM in most of these countries is above 80% according to the reports of international organisations [6]. With an alpha risk of 0.05% for a precision of ± 0.06 percentual units and assuming 10% of non-responders, a sample of 191 subjects is required.

Non responders: a questionnaire will be elaborated for the immigrants selected who refuse to participate in the study. This questionnaire will collect only sociodemographic data, country of origin, etnia, years of residence in Spain and the reason for refusal to participate as well as the name of the person who proposed their participation in the study (interviewer, mediator or both). The aim of this questionnaire is to identify differences between the responder and non responder immigrant populations.

With respect to the healthcare professionals, the different rates of participation in the Primary Healthcare Centres will be taken into account in the analysis. Thus, if necessary, the principal results will be stratified according to the rate of participation.

### Study variables

The questionnaire to the healthcare professionals will assess knowledge on FGM, attitudes and skills in approaching this problem in relation to the following areas:

• Sociodemographic data and of the professional setting: age, gender, professional category, work centre or department.

• Assessment of knowledge on: typology of FGM, cultural origin, geographic distribution and ethnicity of FGM, legal implications, consequences for the health of the women, formation received.

• Assessment of the attitudes of the professionals in relation to: beliefs regarding FGM, professional interest in relation to FGM, convenience or need to act on this problem, fears or insecurity generated on considering this subject, availability of preventive interventions, negativity towards immigrant patients, attitude toward judicial interventions related to FGM and their implications.

• Agreement or disagreement of the professional towards the response or actions to be undertaken in a situation of risk for a girl or towards a recent FGM.

• Previous contact with cases of FGM or situations of risk and previous experiences.

Similarly, the questionnaire for the families will include the following areas:

• Sociodemographic data: age, gender, country of origin, ethnicity, years of residence in Spain, number of children. Position of the different members of the family towards FGM.

• Knowledge of the consequences which FGM may have on the health of a girl.

• Knowledge of Spanish legislation and of the country of origin regarding FGM and the consequences of its practice, experiences (acceptation or rejection) related to the criminalisation of this practice.

• Position (acceptation or rejection) towards a possible preventive action on behalf of the healthcare professionals in Spain.

• Expectations towards the proposal of a preventive intervention aimed at their communities of origin, consequences of not performing FGM in relation to the community in both the country of origin and destination (diaspora), acceptation of alternative rituals to FGM.

Sociodemographic data (age, gender, ethnicity, country of origin, years of residence in Spain and number of children) will be collected from the sub-Saharan healthcare users who do not wish to complete the questionnaire, with the aim of detecting possible differences between users who accept and refuse to participate in the study.

### Questionnaire elaboration

The questionnaires will be developed [29] in several steps described as follows:

**- 1st phase: **Different aspects related to knowledge, attitudes and practices in relation to FGM which may be determined through the questionnaire will be identified from previous experiences, the bibliography consulted and knowledge of the investigative team [14,15]. This will be carried out by the 8 professionals of the investigative team.

**- 2nd phase:, **A group of 8-10 primary care professionals will be selected with the nominal group technique and the most relevant aspects to be incorporated into the questionnaire will be proposed. The nominal group will be composed of primary healthcare professionals with experience in the approach to FGM and providing care to immigrant populations.

**- 3rd phase: **The investigative team will elaborate the questionnaire from the results obtained in the previous phases. The questionnaire to the professionals will be self-administered and the questionnaire to the families will be hetero-administered. This phase will be carried out by the 8 professionals of the investigative team.

**- 4th phase: **The questionnaire will be sent to a multidisciplinary team of primary healthcare professionals (from 20 to 30), randomly selected from the target population, to validate the content and practical aspects of presentation and comprehension of the questions. Between 15 to 20 days after having received the answers, the questionnaire will be resent to the same professionals with the aim of evaluating the stability of the scores. In the case of the families a similar procedure will be carried out with mothers or fathers of girls at an age of risk of undergoing FGM through the help of intercultural mediators.

**- 5th phase: **From the results obtained in the fourth phase, the questionnaire will be modified and the definitive version will be elaborated. This will be carried out by the professionals of the investigative team.

**- 6th phase: **The definitive questionnaire will be administered to the professionals established in the study as well as the families according to the description in the section Study Subjects. A group of responders (from 20 to 30 persons) will be selected to respond once again after 15-30 days with the aim of evaluating the stability of the responses. In the case of the families a similar procedure will be undertaken.

The validity of the construction (correlation between the operative definition of the concept and the aspects evaluated by those surveyed) and the previously described dimensions (validity of content) will be evaluated. The internal consistence of the questionnaire will be analysed with the Cronbach's alpha, both globally and for each of the dimensions. Values between 0.70 and 0.90 will be considered optimum. A factor analysis will be performed to determine the factorial structure of the questionnaire and the agreement between the theoretical dimensions proposed will be evaluated.

Stability will be measured with test-retest concordance, using the simple concordance index and the Kappa coefficient. The results of this index will be evaluated with the Fleiss criteria (< 0.40 poor concordance, 0.40 to 0.75 acceptable concordance and > 0.75 indicates good concordance).

### Data collection

The managing team of each centre or department will be contacted to introduce the study and its objectives. When the administration of the centre authorises participation in the study a member of the research team will go to each of the PHC to inform the professionals, motivate them and invite them to participate. The questionnaires will be given and collected in a specific session in each PHC. One person in each PHC will be responsible for the administration and collection of the questionnaires from the professionals who are absent on the day of the session to be thereafter sent to the research team within a maximum time of two weeks for later processing and analysis of the data collected. The questionnaire to the professionals will be anonymous and self-administered. It will only have a code indicative of the team to which the health professional belongs to obtain the rate of response from each centre. At no time will the data obtained be able to identify the responding professional. Neither will data processing and analysis be able to identify the centres since they will be indexed with an alpha-numerical code with no relationship to the geographic location or service providing entity.

To administer the questionnaires to the families, cultural mediators from the healthcare centres will collaborate. These mediators will introduce the interviewer and will accompany them throughout the whole process of completing the questionnaire to clarify any concept or doubt in relation to the same which may come up among the families surveyed. The participating mediators are experts with specific training in intercultural mediation. For this study they will be instructed as to the characteristics of the questionnaires and the objectives and methods of the study. They will be trained in giving the questionnaire and the objectives sought with each of the questions will be explained. The questionnaire will always be given under the supervision of a member of the investigative team. The questionnaire to the families will be anonymous but hetero-administered. Information regarding the families who refuse to participate in the survey will also be collected.

### Analysis plan

The quality of the data will be evaluated, treating extreme and rare values. Regarding the questionnaire to the healthcare professionals, to answer the first and second objective, each of the issues included in the area of assessment of knowledge (typology of FGM, cultural origin, geographic distribution and ethnicity of FGM, legal implications, consequences for de health of the women and formation recived) and included in the area of assessment of the attitudes of the professionals (beliefs regarding FGM, professional interest in relation to FGM, convenience or need to act on this problem, fears or insecurity generated on considering this subject, availability of preventive interventions, negativity towards immigrant patients, attitude toward judicial interventions related to FGM and their implications) will be described for univariate analysis, mean and standard deviation for quantitative variables and frequency and percentage for qualitative variables. Then a bivariate analysis will be performed between each of the above issues and gender of the healthcare professionals, age, professional category and work centre or department. To do this we will use different tests adequate to the type of data. To answer the third objective, we will do the same as the first and second objective but with each of the issues in the area of agreement or disagreement of the professional towards the response or actions to be undertaken in a situation of risk for a girl or towards a recent FGM. To quantify the frequency of presentation of problems related to FGM in primary care offices (fifth objective), we will do the same as first, second and third objectives but with each of the topics in the area of previous contact with cases of FGM or situations of risk and previous experiences. By analyzing all this information, and mainly from the results obtained with the area of assessment knowledge, we will be able to identify the training needs of the primary care professionals in relation to how to approach FGM, providing a good answer to the fourth objective. A multiple logistic regression model will be used to assess the characteristics of the professionals associated with correct identification of FGM (types that exist, countries in which it is performed, health consequences, reasons for implementation and prosecution by law). Regarding the questionnaire for the families, to describe the sample of immigrant families, each of the issues of the areas of the study will be described for univariate analysis, mean and standard deviation for quantitative variables and frequency and percentage for qualitative variables. To answer the last objective (sixth objective), bivariate analysis will be performed between the area of sociodemographic data and the remaining areas of study to evaluate possible differences between men and women, young persons and the elderly, based on the country of origin, ethnicity, years of residence in Spain and the number of children, with regard to knowledge of the consequences on health which FGM may imply, knowledge of legislation, position towards a possible preventive action on behalf of the healthcare professionals, expectations towards the proposal of a preventive intervention aimed at their communities of origin, consequences of not performing FGM in relation to the community in both the country of origin and destination, and acceptation of alternative rituals to FGM. Comparison between responders and non-responders immigrants will be performed using bivariate analysis to evaluate possible differences in participation between men and women, young and the elderly, based on the country of origin, ethnicity, years of residence in Spain and based on the person who proposed their participation in the study. This will be performed with tests according to the type of distribution and type of variables. Moreover, a multiple logistic regression model will be used to assess the characteristics associated with a possible preventive acceptance by immigrant families. The criterion of statistical significance will be p < 0.05.

### Ethics

This is a questionnaire for professionals who will be verbally informed as to the objectives and content of the questionnaire prior to being interviewed in their work centres. Participation is voluntary. Data analysis will remain totally anonymous to make identification of the participating professionals through their answers impossible. Neither will professionals who decide not to participate be identifiable. No direct intervention will be made (pharmacologic, educative, formative or preventive) in patients or healthcare users or in professionals except for knowing their opinions. The questionnaires for the families will be hetero-administered through a cultural mediator and will not include any information which will allow identification of the person interviewed. The questionnaire will be given in a specific room of the centre to ensure confidentiality and privacy for the participants. Data analysis will remain totally anonymous to make identification of the participating migrant people through their answers impossible. All the databases generated will retain adequate anonymity. Informed consent will be requested from participants in the study. In the case of migrant people, cultural mediators may be required to obtain this informed consent. The management personnel of the centres will be informed of the undertaking of the study and the study has been approved by the ethical committee of the Clinical Research Ethics Committee of the Primary Care Research Institute IDIAP-Jordi Gol (Barcelona, Spain).

## Discussion

The arrival to Spain of families from countries of sub-Saharan Africa in the decade of the 80's, has allowed different cultural realities to be discovered and has shown the presence of traditional practices which are harmful to the health of the girls, daughters of migration, mainly born in Spanish territory but without Spanish nationality. In the case of Spain there is an ethnic reality (mainly from Western Africa), a geographical distribution (basically Catalonia and Aragon) and an accessible number of population which should allow privileged sensitisation [25] since these are communities which are well settled in Spain with strong community links, universalised healthcare and accessability from primary care in all the municipalities of residence.

On the other hand, the growing demographic weight of these communities will no longer make the presence of girls at risk of undergoing FGM exceptional in the near future [30].

We are searching for answers related to whether, when faced with an FGM issue, primary healthcare professionals: a) know and correctly identify the typology of the FGM and its implication for the health of the women, b) know the legal framework and its consequences in Spain, c) have ethical and interventional dilemmas when faced with a hypothetical case of FGM, and d) consider themselves capable of intervening.

Knowledge of the attitudes and needs for trainning of the healthcare professionals of our country, regarding the practice of an emerging health problem such as FGM, should favour the development of strategies of sensitisation and detection of these situations. These strategies could therefore lead to the implementation of educational programmes to develop professional skills in the approach, detection and prevention of this type of situations from the healthcare system to avoid specific legislation related to problems associated with the health and equality of people and thereby make such laws the last step in the chain of prevention.

The approach to this problem raises ethical and legal dilemmas to the primary care professionals related to the consequences of both their intervention as well as non intervention in relation to a suspected case of FGM or the detection of risk for the girls [31-33].

A preventive interdisciplinary task which will allow the designing of strategies focused on the eradication of these practices and evaluation which allows knowledge of the cases produced and the resolution of conflicts considered in both Spain and Africa are therefore essential. Consciencious, reflexive and rigourous work of information, training and sensitisation are necessary. To do this, the proposal herein presented will provide better knowledge of the problem of FGM and how primary care professionals may approach this problem since there are no studies in Spain and very few in Europe. Training strategies and the development of guidelines may therefore be elaborated to enable primary care professionals to prevent and treat this new problem in the PHC.

On the other hand, it may be a model of positive intervention through transcultural care which is respectful of the values and beliefs of the people.

The main limitation of this study is the little collaboration received from the professionals at the time of performing the survey. To surpass this limitation, different researchers of the project will go to the primary healthcare centres to inform the professionals and increase their participation with distribution in a specific meeting with all the professionals of the PHC.

The presence of communities of sub-Saharan immigrants in our area is very concentrated in determined zones of each municipality thereby implying that the level of sensitisation for the problem of the study may vary greatly based on the demographic reality of each PHC. This concentration of immigrant communities in determined areas will not influence the results since our target population is all the professionals regardless of the zone in which they work. There is, therefore, no risk of a biased exploration of this knowledge, attitudes and practices based on the greater or lesser degree of exposure to the study subject. There will obviously be different levels of implication and sensitisation towards the subject which will be shown in the results of the study. With respect to the families, this geographical concentration will facilitate access to the communities although we are aware that there may be social or group conditioners which may influence in the perception of the problem on behalf of the interviewers and in their responses.

The approach to subjects such as FGM, which are closely linked to the identities and cultural backgrounds of the communities, is not easy and thus, we expect to encounter resistance to the determination of the position of the families of the girls at risk. This is a question which has seldom been used externally and is very sensitive to positions which are defensive and closed to the familial-ethnic group setting. Intercultural mediators will help in this task although this will not necessarily guarantee our access to exploring these opinions.

This is a health problem, which is perhaps not very prevalent at present, but which surpasses the purely healthcare framework and confers:

• The violation of the most basic human rights: the right to physical integrity, to health and equality.

• The need for a transcultural approach to these questions which is closely linked to the identity of the migrant persons.

• The moral commitment of the healthcare professionals to avoid these traditional practices which imply a discriminatory, violent, degrading and painful treatment towards women.

Knowledge of these practices and preventive approaches from a transcultural perspective may represent a positive intervention model in relation to integrative care to migrant populations, which is respectful of their values and culture while being effective in the elimination of the physical and psychological consequences.

## Abbreviations

**EU**: European Union; **FGM**: Female Genital Mutilation; **PHC**: Primary Healthcare Centre; **UN**: United Nations Organization; **UNFPA**: United Nations Population Found; **UNICEF**: United Nations Children's Found; **WHO**: World Health Organization.

## Competing interests

The authors declare that they have no competing interests.

## Authors' contributions

JMN, AKM, MJCF, NFR and PTM contributed to formulating the research question, conception and study design. PTM was the editor of the study protocol and contributed to the idea of studying the opinion of the migrant families. AKM, NFR, PTM, JMN, MJCF, EMA and LMO participated in writing the different versions of the questionnaire. AKM, JMN, LMO and PTM revised the last version of the questionnaire. LMO contributed to methodological and statistical support. MRN was in charge of administrative and logistical support. PTM drafted the manuscript. All the authors have read, revised and approved the final manuscript.

## Pre-publication history

The pre-publication history for this paper can be accessed here:

http://www.biomedcentral.com/1471-2458/10/193/prepub
